# Vaccine Efficacy of Self-Assembled Multimeric Protein Scaffold Particles Displaying the Glycoprotein Gn Head Domain of Rift Valley Fever Virus

**DOI:** 10.3390/vaccines9030301

**Published:** 2021-03-23

**Authors:** Paul J. Wichgers Schreur, Mirriam Tacken, Benjamin Gutjahr, Markus Keller, Lucien van Keulen, Jet Kant, Sandra van de Water, Yanyin Lin, Martin Eiden, Melanie Rissmann, Felicitas von Arnim, Rebecca König, Alexander Brix, Catherine Charreyre, Jean-Christophe Audonnet, Martin H. Groschup, Jeroen Kortekaas

**Affiliations:** 1 Department of Virology, Wageningen Bioveterinary Research, 8221 RA Lelystad, The Netherlands; mirriam.tacken@wur.nl (M.T.); lucien.vankeulen@wur.nl (L.v.K.); jet.kant@wur.nl (J.K.); sandra.vandewater@wur.nl (S.v.d.W.); yanyin.lin@hotmail.com (Y.L.); jeroen.kortekaas@wur.nl (J.K.); 2 Institute of Novel and Emerging Infectious Diseases, Friedrich-Loeffler-Institut, 17493 Greifswald-Insel Riems, Germany; Benjamin.Gutjahr@fli.de (B.G.); markus.keller@fli.de (M.K.); Martin.Eiden@fli.de (M.E.); Melanie.Rissmann@fli.de (M.R.); felicitas.var@mail.de (F.v.A.); rebecca.j.koenig@gmail.com (R.K.); Martin.Groschup@fli.de (M.H.G.); 3 Boehringer Ingelheim Veterinary Research Center GmbH & Co. KG, 30559 Hannover, Germany; alexander.brix@boehringer-ingelheim.com; 4 Boehringer Ingelheim, 69007 Lyon, France; catherine.charreyre@boehringer-ingelheim.com (C.C.); Jean-Christophe.AUDONNET@boehringer-ingelheim.com (J.-C.A.); 5 Laboratory of Virology, Wageningen University and Research, 6708 PB Wageningen, The Netherlands

**Keywords:** Rift Valley fever virus, bacterial superglue, multimeric protein scaffold particles, sheep, Gn head domain

## Abstract

Compared to free antigens, antigens immobilized on scaffolds, such as nanoparticles, generally show improved immunogenicity. Conventionally, antigens are conjugated to scaffolds through genetic fusion or chemical conjugation, which may result in impaired assembly or heterogeneous binding and orientation of the antigens. By combining two emerging technologies—i.e., self-assembling multimeric protein scaffold particles (MPSPs) and bacterial superglue—these shortcomings can be overcome and antigens can be bound on particles in their native conformation. In the present work, we assessed whether this technology could improve the immunogenicity of a candidate subunit vaccine against the zoonotic Rift Valley fever virus (RVFV). For this, the head domain of glycoprotein Gn, a known target of neutralizing antibodies, was coupled on various MPSPs to further assess immunogenicity and efficacy in vivo. The results showed that the Gn head domain, when bound to the lumazine synthase-based MPSP, reduced mortality in a lethal mouse model and protected lambs, the most susceptible RVFV target animals, from viremia and clinical signs after immunization. Furthermore, the same subunit coupled to two other MPSPs (*Geobacillus stearothermophilus* E2 or a modified KDPG Aldolase) provided full protection in lambs as well.

## 1. Introduction

Protein-, lipid-, mineral-, and polymer-based scaffolds can be used to increase the immunogenicity of antigens. The coupling of antigens to the surface of these so-called nanoparticles enhances uptake by phagocytic cells, stimulating innate-, humoral-, and cellular immune responses [1]. Importantly, multimeric presentation of antigens enhances the activation of B cells via receptor crosslinking, greatly facilitating humoral immune responses [2,3,4,5,6]. When optimally designed, nanoparticle vaccines can even be efficacious in the absence of adjuvants.

Nanoparticles of different sizes and shapes have been developed, with variable display capacities. Three well-known proteins capable of self-assembly into higher order structures include *Aquifex aeolicus* lumazine synthase (LS) [7], *Geobacillus stearothermophilus* E2 [8], and a modified 2-dehydro-3-deoxy-phosphogluconate aldolase, commonly known as 2-Keto-3-deoxy-6-phosphogluconate (KDPG) aldolase, named I3-01 [9]. All three multimeric protein scaffold particles (MPSPs) require limited post-translational modifications and can be efficiently produced in *E. coli* (or in other expression systems). When the *C*- or *N*-terminal ends of the monomeric proteins in the assembled particle are surface-exposed, this facilitates fusion of antigens to tags or linker modules. Although antigen subunits can also be genetically fused to the *C*- and *N*-terminal ends of the proteins, nanoparticle assembly and integrity could be compromised with increasing structural complexity and size of the antigen. The need for a coordinated folding between the scaffold and the antigen subunit is circumvented when the components are produced separately and subsequently coupled. Chemical conjugation is a generally complex procedure, requiring specific conditions and reagents. Moreover, chemical coupling may result in a heterogeneous decoration of the nanoparticle, compromising efficient presentation to the immune system. Novel technologies are today available that enable highly efficient coupling of antigens to nanoparticles, maintaining their native conformation. A particularly promising technology is referred to as the SpyTag-SpyCatcher “bacterial superglue”, in which a covalent, intermolecular isopeptide bond is formed between the 13 amino acid “SpyTag” peptide and a small (12.3 kDa) “SpyCatcher” protein [10,11]. This two-component superglue is an extremely versatile tool to covalently decorate nanoparticles with antigens and is now being broadly applied to develop vaccines against viral, bacterial and parasitic diseases [12].

As part of the zoonoses anticipation and preparedness initiative project (ZAPI: www.zapi-imi.eu), effective vaccines and antibody therapies are being developed to enable swift response to major new infectious disease threats. The zoonotic Rift Valley fever virus (RVFV) is used as one of the model pathogens to assess the efficacy of the platforms. RVFV is a mosquito-borne bunyavirus (family *Phenuiviridae*, genus *Phlebovirus*) that is pathogenic to ruminants and humans. The virus contains a tri-segmented RNA genome, comprising a small (S), medium (M), and large (L) segment. The S and L segments encode the nucleocapsid and polymerase protein, respectively, involved in genome transcription and replication. The S segment additionally encodes a non-structural protein named NSs, which counteracts host innate immune responses [13]. The M segment encodes a polyprotein precursor protein that is co-translationally cleaved into the two structural glycoproteins Gn and Gc, which are involved in host cell binding and fusion of the viral and endosomal membrane. The M segment additionally encodes a 14-kDa non-structural protein named NSm, which was shown to counteract apoptosis and to be important for dissemination in mosquitoes [14,15,16,17].

The RVFV structural glycoproteins Gn and Gc are the only known targets of neutralizing antibodies (nAbs), and correlate with protection in animal models [18,19]. The elucidation of the cryo-electron microscopy structure of RVFV and the crystal structures of Gn and Gc, followed by the fitting of the protein structures into the T = 12 icosahedral virion, have demonstrated that the Gn head domain (Gn_head_) is fully exposed on the virion surface, explaining that Gn is the dominant target of neutralizing antibodies [20]. However, before these structures were described, we reported the vaccine efficacy of the Gn ectodomain (Gn_head_ region + stem region) produced in insect cells, formulated with Stimune water-in-oil adjuvant, in the mouse and lamb model [21,22]. A single vaccination protected lambs from morbidity and mortality, although low levels of viral RNA were detected after challenge infection, suggestive of low-level viremia [21]. Whereas the latter is of little concern for non-pregnant animals, previous studies have demonstrated that even low-level viremia may result in vertical transmission of the virus in pregnant ewes [23]. A seemingly more efficacious candidate vaccine was developed by co-expression of Gn and Gc in insect cells, resulting in the formation of virus-like particles (VLPs) [22]. This candidate vaccine induced nAbs in mice after a single vaccination, even in the absence of adjuvant. However, the yields of these VLPs seemed insufficient to develop a cost-effective vaccine.

In order to develop a Gn-based vaccine with improved immunogenicity, we assessed in this work the immunogenicity of the Gn_head_ domain of RVFV following coupling of this antigen subunit to the surface of MPSPs. After demonstrating the proof of concept in a murine model, the efficacy of the candidate vaccine was evaluated in the lamb model. Our results show that two vaccinations with adjuvanted MPSPs induces sterile immunity and protects lambs from viremia and clinical signs.

## 2. Materials and Methods

### 2.1. Cells and Viruses

The virulent recombinant RVFV-35/74 strain (recRVFV-35/74) was propagated in BHK-21 cells, which were grown in CO_2_-independent medium (CIM, Gibco), supplemented with 5% fetal calf serum (FCS) (Gibco), 1% L-glutamine (Gibco), and 1% antibiotic/antimycotic (Gibco). For the virus neutralization assay, BHK-21 cells were used, which were grown in GMEM medium, supplemented with 5% FCS, 1% L-glutamine, and 1% antibiotic/antimycotic. Sf9ET cells (ATCC^®^ CRL-3357™) were cultured in Insect-XPRESS medium (Lonza, Maastricht, The Netherlands) supplemented with 1% antibiotic/antimycotic. High Five cells were maintained in an Express Five medium supplemented with 1% glutamine and 1% antibiotic/antimycotic. Both insect cell lines were cultured in suspension at 28 °C in a shaking incubator.

### 2.2. Gn_head_ Antigens Production

DNA fragments encoding the Gn_head_ domain (with or without an *N*-terminal SpyCatcher domain) were flanked by an *N*-terminal GP64 signal sequence and a *C*-terminal twin Strep-Tag (Table 1) and synthesized by GenScript (Piscataway, NJ, USA), resulting in the following open reading frame (ORF) organization: GP64 signal sequence–SpyCatcher–10 GlySer linker–RVFV-Gn_head_–enterokinase site–Twin Strep-tag (Table 1). Following the cloning of the fragment in a pBAC-3 baculovirus transfer vector, recombinant baculoviruses were generated with the flashBAC™ ULTRA baculovirus expression system (Oxford Expression Technologies, Oxford, UK). Briefly, transfection mixtures containing the pBAC-3 vector, a bacmid, and Cellfectin II were added to 24-wells plates containing 200,000 SF9-ET cells per well. Rescued viruses were subsequently amplified in SF9-ET suspension cultures infected at low multiplicity of infection (MOI). For protein production, High Five cells were infected at high MOI according to the manufacturer’s protocol (Thermo Fisher Scientific, Breda, The Netherlands). Proteins were purified from supernatants using Strep-Tactin^®^ resin (IBA, Göttingen, Germany) according to the manufacturer’s instructions. Buffers were exchanged to Tris-buffered saline + 200 mM NaCl using Amicon^®^ Ultra centrifugal filters (Merck-Millipore, Amsterdam, The Netherlands). Proteins were separated in 4–12% SDS gels (Thermo Fisher Scientific, Breda, The Netherlands) and stained with GelCode Blue Stain reagent (Thermo Fisher Scientific, Rockford, IL, USA).

### 2.3. LS-SpyTag Production

*E. coli* expression strain BL21(DE3) was transformed with a pET24a-based vector-plasmid harboring the expression cassette for the LS-SpyTag protein (Table 1). The plasmid was constructed by gene synthesis and *Nde*I and *Sal*I restriction endonuclease-based cloning (Eurofins Genomics, Ebersberg, Germany). The transformed bacteria were grown in auto-induction media (AIM) at 37 °C and 180 RPM in the presence of 30 μg/mL kanamycin for approximately 8 h. Cells were harvested by centrifugation (10,000× *g*, 10 min, 4 °C) and pellets stored at −20 °C until further processing. For recombinant protein purification the cell pellet of 1 L culture was resuspended in 80 mL lysis buffer (50 mM Na Phosphate, 20 mM NaCl, 3 mM ethylenediaminetetraacetic acid (EDTA), pH 7.0) and subjected to 3 freeze-thaw cycles followed by 3 sonication cycles of 7 min each. Soluble and insoluble fractions were separated by centrifugation (15,000× *g*, 30 min, 4 °C) with the insoluble fraction stored at −20 °C until further processing. Subsequently, three washing steps were performed (buffer 1: 50 mM Sodium Phosphate pH 7.0, 2 M NaCl, buffer 2: 50 mM Sodium Phosphate pH 7.0, buffer 3: 2.0% Triton X100, 50 mM sodium phosphate pH 7.0), each followed by a centrifugation step (15,000× *g*, 30 min, 4 °C). The final pellet was solubilized in 50 mM glycine, 8 M Urea, pH 10.0 by overnight incubation with shaking, at 8 °C. Subsequently the solubilized fraction was separated by centrifugation (15,000× *g*, 60 min, 8 °C) and subjected to ion exchange chromatography (IEXC) (Q Sepharose High Performance, buffer A: 50 mM glycine, 8 M Urea, pH 10.0, buffer B: 50 mM glycine, 8 M Urea, pH 10.0, 1 M NaCl). Fractions containing the protein of interest were pooled, diluted twice in buffer (50 mM glycine, 8 M Urea, pH 10.0), dialyzed (molecular weight cut-off) 6–8 kDa, 50 mM Na Phosphate, 150 mM NaCl, pH 7.0), and sterile filtered. Additionally, size-exclusion chromatography (SEC) was performed (Sephacryl S200, buffer A: 50 mM Na Phosphate, 150 mM NaCl, pH 7.0). Relevant fractions were pooled, 10% glycerol added, sterile filtered, aliquoted, and stored frozen (−70 °C).

### 2.4. Aldolase-SpyTag Production

*E. coli* expression strain (BL21(DE3)) was transformed with pET24a-based vector-plasmid harboring the expression cassette for the Aldolase-SpyTag protein (Table 1). The plasmid was constructed via gene synthesis and *Nde*I and *Sal*I restriction endonuclease-based cloning (Eurofins Genomics, Germany). The transformed bacteria were grown in 4 L of auto-induction media (AIM) at 37 °C and 170 RPM in the presence of 30 μg/mL kanamycin. After approximately 4 h, the temperature was reduced to 20 °C and the culture was further incubated overnight (final OD_600_ = 5.3). Cells were harvested by centrifugation (8000× *g*, 20 min, 4 °C) and pellets were stored at −20 °C until further processing. For recombinant protein purification, the cell pellet of 4 L culture was resuspended in a 160 mL lysis buffer (50 mM Na-Citrate, pH 5.5, 20 mM NaCl, 2 mM MgCl_2_, 1 μg/mL Leupeptine, 1 μg/mL Pepstatine, 5 U/mL Benzonase) and submitted to 3 freeze-thaw cycles followed by 3 sonication cycles of 7 min each. The lysate was subsequently clarified by centrifugation (15,000× *g*, 50 min, 4 °C). The supernatant was then subjected to IEXC (SP Sepharose FF, buffer A: 50 mM Na-Citrate, pH 5.5, 20 mM NaCl, buffer B: 50 mM Na Citrate, pH 5.5, 1.0 M NaCl). Fractions containing the protein of interest were pooled and concentrated (approximately 3X, Amicon 100 kDa). Further purification was performed by SEC (S200 Superdex, buffer: 50 mM Tris-HCl pH 8.0, 150 mM NaCl). Relevant fractions were pooled and concentrated (approximately 4X, Amicon 100 kDa). Final material was sterile filtered, aliquoted, and stored frozen in liquid nitrogen.

### 2.5. E2-SpyTag Production

*E. coli* expression strain (BL21(DE3)) was transformed with pET24a-based vector-plasmid harboring the expression cassette for the E2-SpyTag (Table 1). The plasmid was constructed by gene synthesis and *Nde*I and *Sal*I restriction endonuclease based cloning (Eurofins Genomics, Germany). The transformed bacteria were grown in 6 L of auto-induction media (AIM) at 37 °C and 180 RPM in the presence of 30 μg/mL kanamycin for approximately 24 h (final OD_600_ = 4.4). Cells were harvested by centrifugation (8000× *g*, 15 min, 4 °C) and pellets were stored at -20 °C until further processing. For recombinant protein purification the cell pellet of 6 L culture was resuspended in 240 mL lysis buffer (50 mM Na-Citrate, pH 5.5, 20 mM NaCl, 2 mM MgCl_2_, 1 μg/mL leupeptin, 1 μg/mL pepstatine, 5 U/mL benzonase), incubated for 4 °C and subjected to 3 freeze-thaw cycles followed by 3 sonication cycles of 7 min each. The lysate was subsequently clarified by centrifugation (15,000× *g*, 50 min, 4 °C). The supernatant was then subjected to IEXC (Eshmuno S Millipore™, buffer A: 50 mM Na citrate, pH 5.5, 20 mM NaCl, buffer B: 50 mM Na citrate, pH 5.5, 1.0 M NaCl). Fractions containing the protein of interest were pooled and concentrated (approximately 4X, Amicon 100 kDa). Further purification was performed by SEC (S200 Superdex, buffer: 50 mM Tris-HCl pH 8.0, 150 mM NaCl). Relevant fractions were pooled and concentrated (approximately 4X, Amicon 100 kDa). Final material was sterile filtered, aliquoted and stored frozen in liquid nitrogen.

### 2.6. Transmission Electron Microscopy

In total, 5 μL of well-mixed specimen was added to a copper 400 mesh carbon/formvar grid (Electron Microscopy Sciences, Ebersberg, Germany) and incubated for 2 min at room temperature (RT) before being washed with MilliQ (Burlington, MA, USA). Specimen were subsequently stained with 2% phosphotungstic acid (PTA) for 30 s at RT, dried using blotting paper and air, and analyzed in a JEOL 1400Plus transmission electron microscope operated at 120 kV. Images were recorded using a JEOL CCD camera Ruby (8 M pixel).

### 2.7. Experiments with Mice

Six-week-old female BALB/cAnNCrl mice (Charles River Laboratories) were randomly divided into groups of 10 mice, kept in type III filter-top cages under BSL-3 conditions, and allowed to acclimatize for 6 days. Mice were vaccinated intramuscularly (hindleg) at day 0 and 14 with 20 μg Gn_head_ coupled to LS-MPSPs particles in the presence of Stimune, according to the manufactures instructions in 100 μL (Life technologies, Carlsbad, CA, USA). Two weeks after the second vaccination, mice were challenged via intraperitoneal route with 10^3^ TCID_50_ of recRVFV-35/74 [24] in 100 μL medium. Challenged mice were closely monitored and humanely euthanized after reaching a humane endpoint.

### 2.8. Experiments with Lambs

#### 2.8.1. Lamb Trial 1

Conventional 15–17 week-old Texel-German black-headed mutton crossbred lambs, clinically healthy as assessed by a veterinarian, were randomly distributed over 2 groups of 6 animals. After 1 week of acclimatization, lambs in group 1 were vaccinated via intramuscular (IM) route (*M. deltoideus*) with Gn_head_ conjugated to LS (LS:Gn_head_) or 50 μg of unconjugated LS and Gn_head_ (LS + Gn_head_) in 0.33 mL PBS which was formulated with 0.66 mL TS6 adjuvant. A second vaccination with the same antigen, dose, and route was applied two weeks later. Next, 10 days after the second vaccination, all lambs were challenged via the IM route (*M. deltoideus* opposite side to immunization) with 10^5^ TCID_50_ of recRVFV 35/74, which was administrated in 1 mL complete medium. To ensure humane endpoints (HEP) were recognized timely, animals were clinically assessed daily and during critical periods twice per day. Rectal temperatures were determined daily and EDTA and serum blood samples were obtained every second day from half of the animals during the first week after immunization and daily during the first week after challenge, then every second day until the end of the experiment. At the end of the experiment (2 weeks post-challenge), animals were at first sedated with Xylazin (Xylazin 20 mg/mL, CP-Pharma Handelsgesellschaft mbH, Burgdorf, Germany) and finally euthanized with a combination of embutramid, tetracain hydrochloride, and mebezoniumiodid (T61, MSD, Kenilworth, NJ, USA) according to the manufacturer’s instructions. Plasma samples were analyzed for the presence of RVFV RNA with quantitative reverse-transcription PCR (RT-qPCR).

#### 2.8.2. Lamb Trial 2

Conventional 8–10 week-old Texel-Swifter crossbred lambs, clinically healthy as assessed by a veterinarian, were randomly distributed over four groups of 8 animals. After 1 week of acclimatization, lambs of groups 1–3 were vaccinated via IM route (*biceps femoris*) with 50 μg RVFV-Gn_head_ conjugated to LS, E2, or Aldolase MPSPs in 0.33 mL TBS, which was formulated with 0.66 mL TS6 adjuvant. A second vaccination with the same dose and route was applied 2 weeks later. Lambs in group 4 were mock-vaccinated and also served as a control group for another vaccine trial [25]. Two weeks after the second vaccination, all lambs were challenged via the IV route (*jugular* vein) with 10^5^ TCID_50_ of recRVFV 35/74, which was administrated in a 1 mL complete CIM medium. To ensure HEPs were recognized timely, animals were clinically assessed daily and during critical periods, two or three times per day. Rectal temperatures were determined daily and serum blood samples were obtained weekly. EDTA blood samples were taken weekly but during the first 6 days post-vaccination and 11 days post-challenge, additional daily EDTA blood samples were taken. At the end of the experiment (2 weeks post-challenge), animals were euthanized via exsanguination after being anesthetized with 50 mg/kg sodium pentobarbital (Euthasol^®^, ASTfarma BV, Oudewater, The Netherlands) applied via the IV route. Plasma samples were analyzed for the presence of RVFV RNA via RT-qPCR.

### 2.9. Preparation of Organ Suspensions

Ten % organ homogenates were prepared using the ULTRA-TURRAX system in combination with DT-20 tubes (IKA, Staufen, Germany). Briefly, 0.6 g tissue  was homogenized in 6 mL culture medium for 40 s followed by removal of cell debris by slow-speed centrifugation. The suspensions were used for virus detection by RT-qPCR and virus isolation.

### 2.10. RNA Isolation and RT-qPCR

Viral RNA was isolated with the NucliSENS easyMAG system, according to the manufacturer’s instructions (bioMerieux, France), from either 0.5 mL of plasma or 0.5 mL of 10% organ suspension. Briefly, 5 μL RNA was used in a RVFV RT-qPCR using the LightCycler one-tube RNA Amplification Kit HybProbe (Roche, Almere, The Netherlands) in combination with a LightCycler 480 real-time PCR system (Roche) and the RVS forward primers (AAAGGAACAATGGACTCTGGTCA), the RVAs (CACTTCTTACTACCATGTCCTCCAAT) reverse primer, and a fluorescein amidite (FAM)-labelled probe RVP (AAAGCTTTGATATCTCTCAGTGCCCCAA) [21]. Virus isolation was performed on RT-qPCR positive samples with a threshold above 10^5^ RNA copies/mL as this has been previously shown to be a cut-off point below which no live virus can be isolated.

### 2.11. Serology

A virus neutralization test was performed as described [26], using a RVFV-4s variant encoding green fluorescent protein (RVFV-4s__GFP_) [27]. Briefly, three-fold serial dilutions of inactivated (2 h at 56 °C) sera were mixed with a fixed amount of virus (~200 TCID_50_) in 96-wells plates. After a 2 h incubation period, 20,000 BHK-21 cells were added to each well and plates were incubated for 2 days at 37 °C and 5% CO_2_, followed by evaluation of GFP expression. Neutralizing titers were determined using the Spearman-Kärber algorithm.

A RVFV-Gn_head_ specific indirect ELISA was performed by coating ELISA plates (Greiner 655092) with 0.5 μg/mL RVFV-Gn_head_ in a coating buffer (pH 9.6, WBVR, 100 μL/well). Following overnight incubation at 4 °C, plates were washed with PBS supplemented with 0.05% Tween 20 (PBST_20_) using an ELISA washer (6 pulses). Plates were blocked with 200 μL/well of blocking buffer (PBS + 0.05% Tween 20 + 2% fresh-made skimmed milk) and incubated for 1h at RT. Plates were subsequently incubated with 100 μL/well of five-fold dilution series of sera samples in blocking buffer (starting at a dilution of 250×) for 1 h at RT and then washed with the ELISA washer. Horseradish peroxidase (HRP)-conjugated rabbit anti-sheep IgG antibody (ab6747, Abcam) in the blocking buffer was used as a secondary antibody (100 μL/well) for 1 h at RT. After a final washing step with the plate washer, 100 μL/well of BioFX^®^ TMB One Component HPR Microwell Substrate (TMBW-1000-01, SurModics, Eden Prairie, MN, USA) was added as a substrate, followed by incubation for 10 min at RT. The reaction was stopped by adding 50 μL/well of 0.5 M H_2_SO_4_. Optical densities were measured at OD_450_ nm with a Mutiskan RC plate reader (Thermo Labsystems, Philadelphia, PA, USA) using Ascent Software Version 2.6 (Thermo Scientific, Waltham, MA, USA).

### 2.12. Statistical Analysis

Data were statistically analyzed using the Mann–Whitney test in GraphPad Prism. *P* values < 0.05 were taken as significant. Data were expressed as means with SD.

## 3. Results

### 3.1. Production of SpyCatcher-RVFV-Gn_head_ and SpyTag-MPSPs

**Figure 1:**
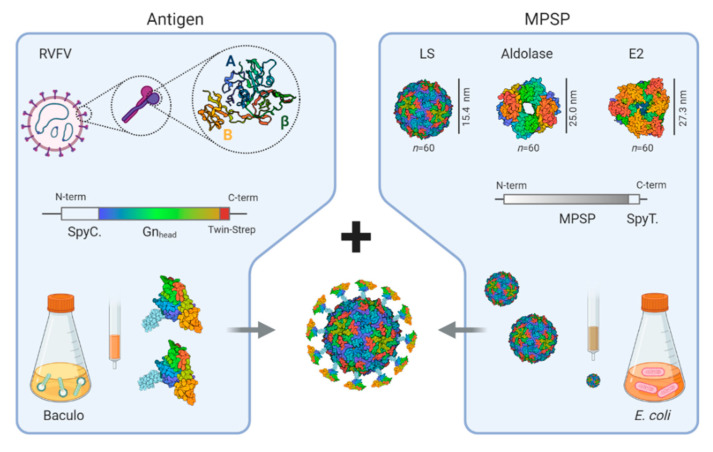
Graphical presentation of the multimeric protein scaffold particles (MPSPs)-based Rift Valley fever virus (RVFV) candidate vaccines. The RVFV-Gn_head_ domain comprising the A (I), β (II), and B (III) subdomain was *N*-terminally linked to a SpyCatcher domain and a *C*-terminal Twin-Strep-tag. Following expression using the baculovirus expression system, the subdomains were purified by Strep-Tactin column chromatography. Lumazine synthase (LS)-, Aldolase-, and E2-based MPSPs with *C*-terminal SpyTags were expressed in *E. coli*. Upon mixing, the SpyCatcher, fused to the *N*-terminus of Gn_head_ forms a spontaneous isopeptide bond with the SpyTag present on the MPSPs, yielding antigen-decorated nanoparticles for immunization.

**Figure 2:**
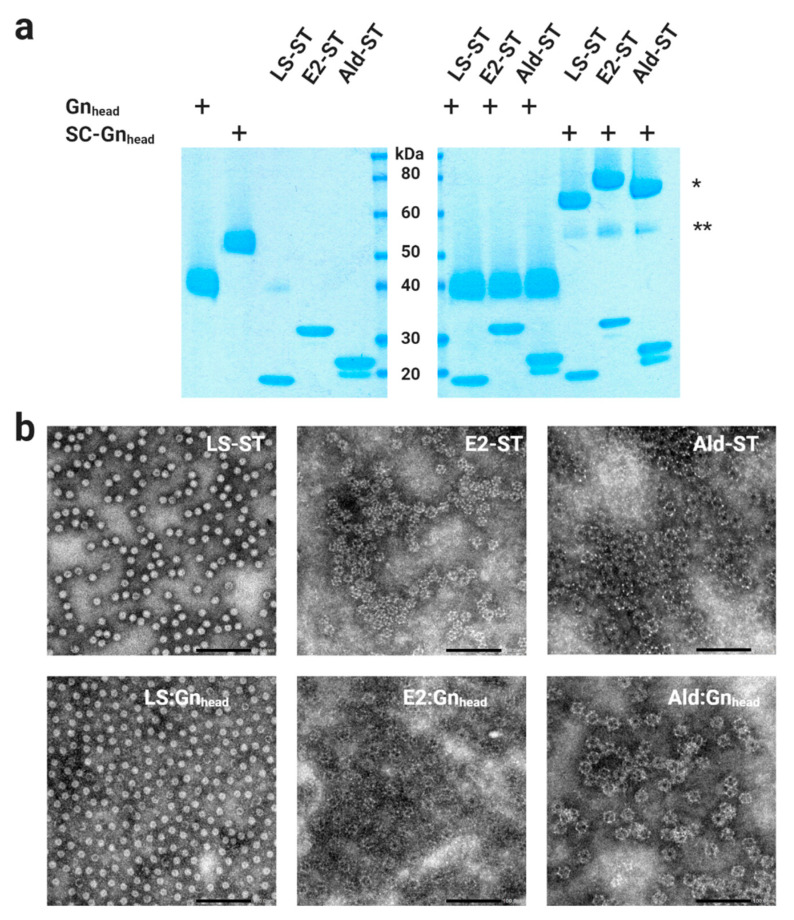
Confirmation of RVFV-Gn_head_ coupling to MPSPs. (**a**) SDS-PAGE of purified Gn_head_ and SpyCatcher-linked Gn_head_; SC-Gn_head_. Purified MPSPs with *C*-terminally-linked SpyTags (ST) and LS-ST, E2-ST, and Ald-ST MPSPs mixed with either Gn_head_ or SC-Gn_head_ for 1 h at room temperature (RT) at a ratio just above particle saturation. Coupled MPSP-derived monomers (*) and remaining free antigen (**) is indicated. The lowest band in the Ald-ST lanes most likely represents a proteolytic degradation product. The plus sign means that Gn_head_ or SC-Gn_head_ was present in the sample (**b**) TEM images (negative stain) of the SpyTag-linked MPSPs with or without conjugated SC-Gn_head_ antigen. Particles were uniformly dispersed and no evidence of aggregation was observed. The colon (:) represents coupling of antigen to MPSPs. Scale bar: 100 nm.

The Gn_head_ domain is located at the virion surface, shielding the Gc protein [20], and is the main target of neutralizing antibodies [21,28,29,30,31]. The Gn_head_ domain consists of three subdomains: domain A (also called domain I), β-ribbon (domain II), and domain B (domain III) [20,30,32]. Here, we genetically fused a SpyCatcher domain to the *N*-terminus of Gn_head_ (SC-Gn_head_) to facilitate conjugation to scaffolds displaying SpyTags, and additionally a *C*-terminal Twin-Strep-tag to facilitate purification (Figure 1). The fusion protein was expressed with the baculovirus expression system. As a control for conjugation experiments, the Gn_head_ domain was also expressed without a SpyCatcher domain. Both the Gn_head_ domain with and without the SpyCatcher were produced at a small scale in around 10–20 mg/L of a culture medium of baculovirus-infected High Five cells. Purity was analyzed via sodium dodecyl sulfate polyacrylamide gel electrophoresis (SDS-PAGE) and proteins were found to be of the expected sizes (Figure 2a).

Three different scaffold proteins were produced as well; all three self-assembled as 60 monomers into MPSPs of different sizes (Figure 1). The first MPSP is based on *Aquifex aeolicus* lumazine synthase (LS), a 20-kDa protein that assembles via 12 pentamers into an icosahedral particle of 15 nm. The second nanocarrier is based on I3-01 (I3), a modified KDPG aldolase of 24-kDa, that assembles into a 25 nm dodecahedron. The third nanocarrier is based on *Geobacillus stearothermophilus* E2 of 26-kDa, which assembles into a pentagonal dodecahedral scaffold of 27 nm. All three MPSPs were produced in *E. coli* with a *C*-terminal SpyTag (Figure 1). Small scale production resulted in yields, after purification from *E. coli* fermentation pellets, of approximately 80, 30, and 20 mg/L, respectively, for LS, E2, and Aldolase. Purity was analyzed via SDS-PAGE and proteins were found to be of the expected sizes (Figure 2a).

### 3.2. Coupling of the RVFV-Gn_head_ Subunit Antigen to the MPSPs

In order to bind the SpyCatcher-Gn_head_ domain subunit to the MPSPs displaying SpyTags, the purified subunit was simply mixed with the different MPSPs, and incubated at RT for 1 h, followed by quality assessment via SDS-PAGE (Figure 2a) and transmission electron microscopy (TEM, Figure 2b). As a control, we took along Gn_head_ lacking the SpyCatcher domain. Optimal coupling, defined as achieving antigen-saturated particles, were obtained after mixing MPSP monomers with SC-Gn_head_ at a molar ratio of 1:2.6 for LS, 1:4 for Aldolase, and 1:3.9 for E2 (Figure 2). The TEM analysis of unconjugated particles subsequently showed particle morphologies in line with literature [33,34,35], and morphology was not visibly compromised by the coupling of antigen to the MPSPs.

### 3.3. Coupling to MPSPs Results in Improved Efficacy in a Lethal RVFV Mouse Model

**Figure 3:**
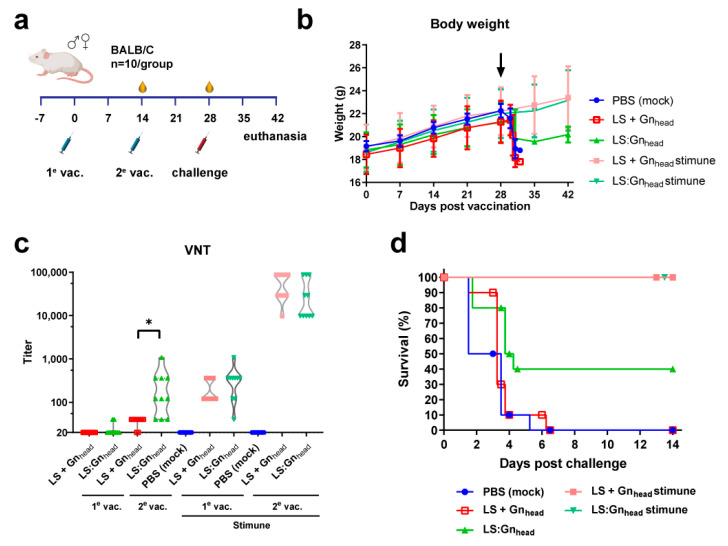
RVFV vaccination-challenge experiment with Gn_head_-conjugated LS particles in mice. (**a**) Schematic presentation of the experimental setup. (**b**) Average body weights with SD following vaccination (day 0 and 14) and challenge (day 28; black arrow). (**c**) Neutralizing antibodies in serum following the first (day 14) and second vaccination (day 28). The x-axis is set to the limit of detection of the VNT. (**d**) Survival of vaccinated animals following RVFV challenge.

To assess the in vivo efficacy of MPSP-Gn_head_, groups of 10 mice were immunized twice, at a two-week interval, with either 20 μg of SC-Gn_head_ coupled to LS-ST (LS:Gn_head_) or 20 μg of Gn_head_ combined with 20 μg LS-ST (without coupling: LS + Gn_head_) in the presence or absence of Stimune water-in-oil adjuvant (Figure 3a). Vaccine doses were standardized based on the amount of Gn_head_. Two weeks after the second vaccination, the mice were challenged via intraperitoneal route with 10^3^ TCID_50_ of recRVFV-35/74. No injection site reactions or other untoward effects such as weight loss were noted following vaccination (Figure 3b). A RVF virus neutralization test (VNT) [36] revealed that following the second vaccination in the absence of Stimune, significantly higher neutralizing antibody titers were observed in the group vaccinated with LS:Gn_head_ as compared to the group vaccinated with unconjugated LS and Gn_head_ (Figure 3c). The observed VNT responses correlated with the survival rates following challenge. All mice succumbed to the infection or had to be euthanized after reaching a HEP within 5 days post challenge in the LS + Gn_head_ group, whereas 40% of mice survived the challenge in the LS:Gn_head_ group. In the presence of Stimune, all mice developed high VNT responses and survived the challenge, irrespective of coupling (Figure 3d).

### 3.4. Coupled Particles Show Improved Efficacy in the RVFV Lamb Model

**Figure 4:**
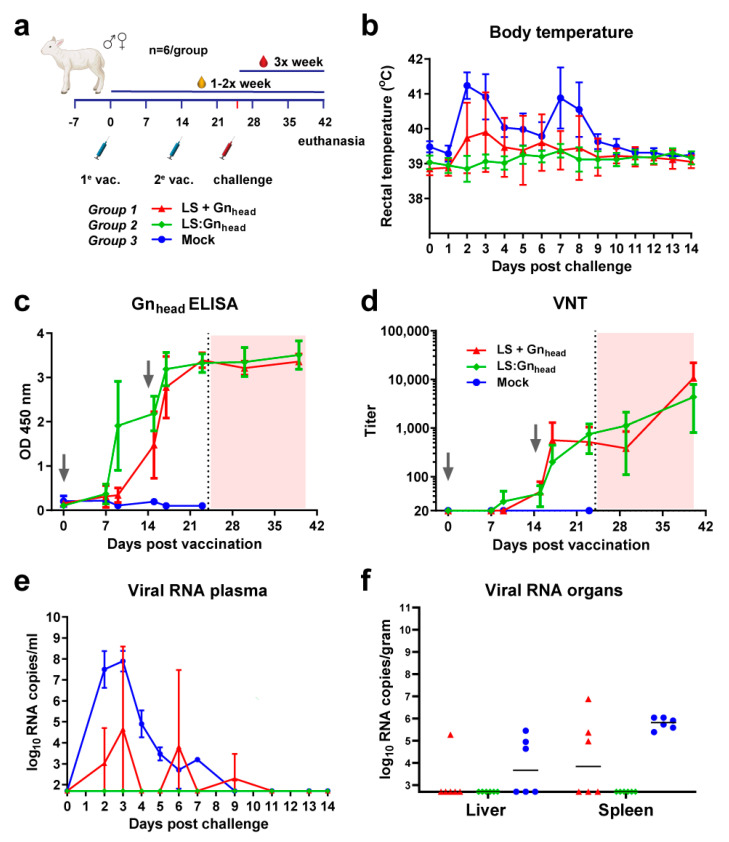
RVFV vaccination-challenge experiment with Gn_head_-conjugated LS particles in lambs. (**a**) Schematic presentation of the vaccination regimen and vaccines. (**b**) Average rectal temperatures of vaccinated and mock-vaccinated lambs post RVFV challenge. (**c**) Gn_head_-specific antibody responses as determined by ELISA. (**d**) Neutralizing antibody responses in weekly-obtained serum samples. The x-axis is set to the limit of detection of the VNT and wells were considered positive when >50% inhibition of viral growth was observed. (**e**) Monitoring of viral RNA in vaccinated and mock-vaccinated lambs using RT-qPCR. Samples that tested negative are depicted at the detection limit of the PCR (1.3 log_10_ RNA copies/mL) (**f**) Viral RNA in liver and spleen samples of vaccinated and mock-vaccinated lambs using RT-qPCR. Samples that tested negative are depicted at the detection limit of the PCR (2.3 log_10_ RNA copies/mL). No animals succumbed or reached a HEP in this experiment. Error bars in panels b, c, d and e represent SDs.

After demonstrating vaccine efficacy in the mouse model, we evaluated the efficacy of LS:Gn_head_ versus LS + Gn_head_ in lambs, the most susceptible natural target species. As the results obtained with mice suggested that adjuvant is required to achieve complete protection, TS6 adjuvant, suitable for use in ruminants, was used. TS6 is an oil-in-water (O/W) emulsion that comprises in the oily-phase sorbitan monooleate, sorbitan trioleate, paraffin oil, and sodium mercurothiolate, monopotassium, and disodium phosphate in the aqueous phase. Lambs were immunized twice at a 14 day interval with either 50 μg of Gn_head_ conjugated to LS (LS:Gn_head_) or 50 μg of unconjugated LS and Gn_head_ (LS + Gn_head_). They were challenged 10 days after the second vaccination. The serological analysis revealed that VNT responses did not differ significantly between the LS:Gn_head_ and LS + Gn_head_ group before the challenge. However, complete protection from the RVFV challenge, as evidenced by prevention of pyrexia and viremia, was only achieved in the LS:Gn_head_ group (Figure 4).

### 3.5. The Type of MPSP Evaluated Does Not Determine Immunogenicity and Vaccine Efficacy

**Figure 5:**
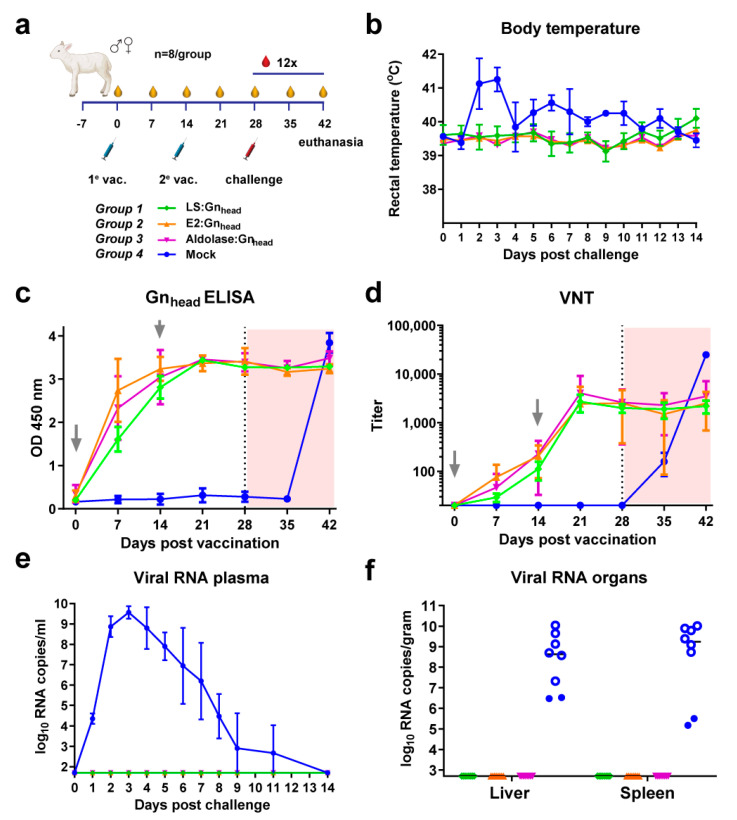
RVFV vaccination-challenge experiment with Gn_head_-coupled MPSP particles in lambs. (**a**) Schematic presentation of the vaccination regimen and vaccines. (**b**) Average rectal temperatures of vaccinated and mock-vaccinated lambs post RVFV challenge. (**c**) Gn_head_-specific antibody responses as determined by ELISA. (**d**) Neutralizing antibody responses in weekly-obtained serum samples. The x-axis is set to the limit of detection of the VNT and wells were considered positive when >50% inhibition of viral growth was observed. (**e**) Monitoring of viral RNA in vaccinated and mock-vaccinated lambs by RT-qPCR. Samples that tested negative are depicted at the detection limit of the PCR (1.3 log_10_ RNA copies/mL) (**f**) Viral RNA in liver and spleen samples of vaccinated and mock-vaccinated lambs by RT-qPCR. Samples that tested negative are depicted at the detection limit of the PCR (2.3 log_10_ RNA copies/mL). Open symbols indicate animals that succumbed or were euthanized due to the challenge infection prior to study end. Error bars in panels b, c, d, and e indicate SDs.

To assess whether the MPSP used as a scaffold influences immunogenicity and vaccine efficacy, we compared the efficacy of LS:Gn_head_, E2:Gn_head_, and Aldolase:Gn_head_ head-to-head in another lamb vaccination-challenge trial, again formulated with TS6 adjuvant (Figure 5). Lambs were immunized twice, with a two-week interval with 50 μg of Gn_head_-conjugated MPSPs followed by RVFV challenge two weeks after the second vaccination (Figure 5a). The Gn_head_ ELISA and VNT results showed highly similar and robust immune responses in all animals, irrespective of the type of MPSP used. Following challenge, all vaccinated animals were protected with complete absence of viremia and clinical signs, whereas all mock-vaccinated animals developed high viremia with associated temperature increases (Figure 5). These results suggest that the type of the MPSP used in this experiment is not a key determinant in the induction of protective immune responses.

## 4. Discussion

We reported the vaccine efficacy of a MPSP-based RVF vaccine in mice and lambs. Protection was correlated with the induction of neutralizing antibodies in both species, which were boosted by a second vaccination. The antigen coupled MPSP vaccine complex was shown to induce superior immune responses and protection in both mice and lambs compared to the unconjugated variants. No significant differences were observed in immune responses between lambs vaccinated with the same antigen coupled to different MPSPs.

Although we observed a significant beneficial effect of coupling in our mouse experiment for the induction of neutralizing antibody responses in the absence of the adjuvant, immune responses in the presence of adjuvant (Stimune) were highly similar and all animals were protected. To assess the potential beneficial effect of coupling in the RVFV mouse model in more detail, a dose-titration experiment with coupled versus uncoupled antigen in the presence and absence of adjuvant should be conducted. Interestingly, for other targets, including SARS-CoV-2, vaccination with relatively low amounts of nanoparticle-coupled antigen showed promise [37,38,39,40,41,42]. The observed full protection in the presence of adjuvant also highlights that Gn_head_ is a potent immunogen, possibly even more potent than the complete Gn ectodomain [21,43,44].

Considering that ruminants are the most susceptible target animals of RVFV, we subsequently assessed whether vaccination with free Gn_head_ antigen or Gn_head_ coupled to MPSPs was efficacious in the lamb model. In the lamb trials, we made use of the TS6 O/W adjuvant. In the first lamb trial, complete protection from viremia and clinical signs was achieved, which depended on coupling to MPSPs. Despite the clear difference in vaccine efficacy between the two groups, neutralizing antibody levels did not significantly differ at the moment of challenge virus administration. This suggests that cellular immune responses contributed to vaccine efficacy, especially in animals vaccinated with antigen coupled to MPSPs. The induction of cellular immune responses following nanoparticle immunization is expected to be mediated by cytotoxic T-cells via major histocompatibility complex (MHC) cross-presentation [12,45,46].

In the second lamb trial, we assessed whether the type of the MPSP is an important determinant of vaccine immunogenicity and efficacy. As we did not observe significant differences in antibody responses and vaccine efficacy, we proposed prioritization among these three MPSPs based on large scale manufacturing yields, easiness of downstream purification, stability, and costs of goods. The results so far suggest that MPSPs E2 and I3-03 hold more promise. However, to conclude that the type of MPSP is a minor determinant with respect to immunogenicity, additional head-to-head comparisons are needed. Of note, the three MPSPs used in this study each result from assembling of monomeric scaffold proteins into 15–30 nm particles with 60 sites for antigen conjugation.

Besides the evaluation of additional MPSPs, several other optimizations could further improve the methodology. We here fused the SpyCatcher domain to the *N*-terminus of Gn_head_, whereas fusion to the *C*-terminus of Gn_head_ may have resulted in a more optimal presentation of the Gn subunit. Domain A (I) and domain B (III) were considered the dominant targets of neutralizing antibodies in a virion context [30,32,47]. Whether the individual subdomains A (I) and B (III) are potent immunogens capable of inducing protective immunity awaits further study. In general, production yields of smaller antigens is superior to large antigens and smaller antigens are expected to face less spatial constrains with regard to conjugation to the MPSPs. However, from a veterinary vaccine perspective, we note that, in the current setup, a double vaccination regimen has to be used, which does not yet compete with live-attenuated vaccines or vector-based vaccines providing single-shot immunity [25] and data on long-term memory responses is still lacking. Nevertheless, further construct optimization combined with the use of novel production systems like *Myceliophthora thermophila*, also referred to as C1 [48], is likely to result in an efficacious and cost-effective MPSP-based RVF vaccine.

## 5. Conclusions

The results of this study show that a double vaccination with the Gn_head_ domain, when bound to lumazine synthase-based MPSPs, prevents mortality in a lethal mouse model and protects lambs, the most susceptible RVFV target animals, from viremia and clinical signs. Furthermore, similar vaccination with the same subunit coupled to two other MPSPs provides complete protection in lambs as well.

## Figures and Tables

**Table 1:** Amino acid sequences of antigens, MPSPs, superglue domains and tags.

Protein Domain	Amino Acid Sequence
GP64 signal sequence	MPMLSAIVLYVLLAAAAHSAFA
SpyCatcher	DIPTTENLYFQGAMVDTLSGLSSE QGQSGDMTIEEDSATHIKFSKRD EDGKELAGATMELRDSSGKTIS TWISDGQVKDFYLYPGKYTFVETAAPDGYEV ATAITFTVNEQGQVTVNGKATKGDA HIDGPQGIWGQLEWKK
10 GlySer linker	GGGGSGGGGS
RVFV-Gn_head_	EDPHLRNRPGKGHNYIDGMTQEDATCK PVTYAGACSSFDVLLEKGKFPLFQSYAHHRTL LEAVHDTIIAKADPP SCDLQSAHGNPCMKEKLVMKTHCPNDYQSA HYLNNDGKMASVKCPPKYELT EDCNFCRQMTGASLKKGSYPLQDLFCQSSED DGSKLKTKMKGVCEVGVQAL KKCDGQLSTAHEVVPFAVFKNSKKVYLDKLD LKTEENLL PDSFVCFEHKGQYKGTMDSGQTKRELKSFDIS QCPKIGGHGSKKCTG DAAFCSAYECTAQYANAYCSHANGSGVVQI QVSGVWKKPLCVGYERVVVKRE
Entero Kinase site	DDDDK
Twin Strep-tag	GSAWSHPQFEKGGGSGGGSGGSAWSHPQFEK
LS-SpyTag ^1^	MQIYEGKLTAEGLRFGIVASRFN HALVDRLVEGAIDAIVRHGGREEDITLVRVPG SWEIPVAAGELARKEDIDAVIAIGVLIRGATPH FDYIASEVSKGLANLSLELRKPITFGVIT ADTLEQAIERAGTKHGNKGWEAALSAIE MANLFKSLRGGGGSGGGGSGGGGS **AHIVMVDAYKPTK**
Aldolase-SpyTag ^1^	**MAHIVMVDAYKPTK**LINGGSGGSGGSGGSM KMEELFKKHKIVAVLRANS VEEAKKKALAVFLGGVHLIEITFTVPDADTVIK ELSFLKEMGAIIGAGTV TSVEQCRKAVESGAEFIVSPHLDEE ISQFCKEKGVFYMPGVMTPTELVKAMKLGHTI LKLFPGEVVGPQFV KAMKGPFPNVKFVPTGGVNLDNVCEW FKAGVLAVGVG SALVKGTPVEVAEKAKAFVEKIRGCTE
E2-Spytag ^1^	**MAHIVMVDAYKPTK**AAAEEKAAPAAAK PATTEGEFPETREKMSGIRRAIAKAMVH SKHTAPHVTLMDEADVTKLVAHRKKFKAIAA EKGIKLTFLPYVVKALVSALREYPVLNTSID DETEEIIQKHYYNIGIAADT DRGLLVPVIKHADRKPIFALAQEINELAEKAR DGKLTPGEMKGASCTIT NIGSAGGQWFTPVINHPEVAILGIGRIAEK PIVRDGEIVAAP MLALSLSFDHRMIDGATAQKALNHIKRLLSD PELLLMEA

^1^ The SpyTag sequence is presented in bold.
